# Mucosal Therapy of Multi-Drug Resistant Tuberculosis With IgA and Interferon-γ

**DOI:** 10.3389/fimmu.2020.582833

**Published:** 2020-10-20

**Authors:** Andy C. Tran, Gil R. Diogo, Matthew J. Paul, Alastair Copland, Peter Hart, Nickita Mehta, Edward. B. Irvine, Tufária Mussá, Pascal M. W. Drake, Juraj Ivanyi, Galit Alter, Rajko Reljic

**Affiliations:** ^1^ Institute for Infection and Immunity, St. George’s University, London, United Kingdom; ^2^ Ragon Institute, Harvard, Cambridge, MA, United States; ^3^ Department of Microbiology, Faculty of Medicine, Eduardo Mondlane University, Maputo, Mozambique; ^4^ Departamento de Plataformas Tecnológicas em Saúde, Instituto Nacional de Saúde, Maputo, Mozambique; ^5^ Guy’s Campus of King’s College London, London, United Kingdom

**Keywords:** tuberculosis, IgA, mAb—monoclonal antibody, drug resistance, HspX, alpha crystallin, CD89 (Fc alpha/muR), interferon-gamma

## Abstract

New evidence has been emerging that antibodies can be protective in various experimental models of tuberculosis. Here, we report on protection against multidrug-resistant *Mycobacterium tuberculosis* (MDR-TB) infection using a combination of the human monoclonal IgA 2E9 antibody against the alpha-crystallin (Acr, HspX) antigen and mouse interferon-gamma in mice transgenic for the human IgA receptor, CD89. The effect of the combined mucosal IgA and IFN-γ; treatment was strongest (50-fold reduction) when therapy was applied at the time of infection, but a statistically significant reduction of lung bacterial load was observed even when the therapy was initiated once the infection had already been established. The protection involving enhanced phagocytosis and then neutrophil mediated killing of infected cells was IgA isotype mediated, because treatment with an IgG version of 2E9 antibody was not effective in human IgG receptor CD64 transgenic mice. The Acr antigen specificity of IgA antibodies for protection in humans has been indicated by their elevated serum levels in latent tuberculosis unlike the lack of IgA antibodies against the virulence-associated MPT64 antigen. Our results represent the first evidence for potential translation of mucosal immunotherapy for the management of MDR-TB.

## Introduction

The evidence for a protective role of antibodies in tuberculosis (TB) has been strengthened significantly over the last decade or so, owing to a number of recent reports (1–5). The common theme in these reports is that under certain experimental conditions, some antibodies can exert protective activity while others cannot, and this may depend on several factors including their origin, isotype, and specificity, as well as posttranslational modifications. Thus, Li et al. (2) showed that polyclonal antisera from some healthcare workers exposed to *Mycobacterium tuberculosis* who were latently infected or uninfected, exerted a protective effect when transferred to mice prior to challenge with *M. tuberculosis*. Furthermore, the same study reported that antibodies from healthcare workers were more protective than those from TB patients, and this appears to be a common theme emerging from the most recent studies. Thus, Lu et al. (3), also reported that antibodies from individuals with latent *M. tuberculosis* infection were superior to those from active TB patients in their capacity to mediate intracellular killing of the bacteria by macrophages. This was associated with distinct glycosylation profiles of these antibodies which conferred superior functional properties and in particular enhanced interaction with the immunoglobulin Fc receptors on phagocytic cells.

In addition to their specificity, another feature of antibodies that may influence their function in *M. tuberculosis* infection is their isotype. This was shown, when intranasal delivery of murine IgA, but not IgG monoclonal antibodies (mAb) of the same alpha crystalin (Acr) antigen specificity, when combined with mouse interferon-γ, imparted protection against *M. tuberculosis* lung infection in mice (6, 7) and could also prevent post-chemotherapy relapse of infection and lung pathology (6–9). The role of the IgA isotype was further supported by demonstrating protection using a human IgA mAb in CD89 human IgA-receptor transgenic mice (10). Recently, the role of IgA isotype was shown by the finding under *in vitro* conditions, that IgA antibodies specific for lipoarabinomannan (LAM) and heparin-binding hemagglutinin adhesin (HBHA) were bacteriostatic whereas IgG antibodies exacerbated infection (5). This, however, may be epitope dependent, since several other studies reported protective effects of various IgG antibodies (11–14).

The synergy between the murine anti-Acr mAb TBA61 and mouse IFN-γ was previously demonstrated following their intranasal inoculation, by better protection against intranasal *Mtb* challenge and by bactericidal action and increased nitric oxide and TNF production by mouse peritoneal macrophages *in vitro* (8). The mechanisms involving both Fcα-receptor and IFN-γ mediated macrophage functions were possibly related to the IgA and IFN-γ synergistic inhibition of the growth of J774 mouse macrophage cell lines and the induction of TNF synthesis and apoptosis of mouse peritoneal exudate macrophages (8). Furthermore, we have previously shown that while IFN-γ had some inhibitory effect on *Mtb* infection in CD89 transgenic mice, the greatest protective effect was seen when mice were treated with a combination of both 2E9IgA and IFN-γ (10). A synergy between antibody and IFN-γ action *in vitro* was also reported for another intracellular pathogen, *Brucella melitensis* (15). Other possible mechanisms involving the IgA binding to the Gal-3 (Mac-2) intracellular lectin were also considered (16).

In the current study, we set out to explore the effectiveness of combined IgA and IFN-γ; immunotherapy (CIT) against MDR-TB infection as the key step toward potential translation of this approach for clinical application. MDR-TB is currently on the rise and poses a significant challenge to the healthcare systems around the world, but particularly in the developing countries, which shoulder the biggest burden of both TB and MDR-TB. While the new drugs such as bedaquiline (17) and delamanid (18) hold promise, treatment of MDR-TB still remains difficult, with long duration, significant drug toxicity and comparatively inferior cure rates, all being unsatisfactory. An adjunct form of immunotherapy such as CIT would therefore be highly desirable against MDR-TB and also other resistant infections, as it could improve these outcomes by acting on the ‘persister’ bacteria resisting drug treatment, thus shortening the treatment duration and improving the cure rates (19).

Using a transgenic mouse model expressing either human IgA (CD89) or IgG (CD64) receptors (20), we demonstrate effectiveness of IgA but not IgG and elucidate potential mechanisms involved in protection. The specificity of the 2E9IgA1 mAb appears to be important, since its antigenic target, the alpha crystallin (Acr, HspX, 16 kDa) was shown to be expressed on the surface of mycobacteria, thus facilitating 2E9IgA1 mediated phagocytic uptake. Finally, we detected similar levels of anti-Acr IgA antibodies not only in sera of TB patients but, surprisingly, also of latently infected individuals (LTBI) and healthy BCG vaccinated controls, suggesting a potential role for IgA and Acr in the control of *M. tuberculosis* infection.

## Materials and Methods

### Purification and Biophysical Characterization of 2E9IgA1

Expression of 2E9IgA1 mAb in Chinese Hamster Ovary (CHO) cells was previously described (10). To purify the antibody, transfected CHO cells were grown in CellSTACK culture flasks (Corning, Deeside, UK) kept at 37°C 5% CO_2_ to confluency in DMEM medium (Sigma, Poole, UK) supplemented with 10% w/v FBS (Sigma), 2 mM L-glutamine (Sigma), 100 units/ml of penicillin, and 100 µg/ml of streptomycin (Sigma). Cell culture supernatants were harvested, filter sterilized using 0.22 µm PES filters (Millex, Sigma), and subjected to affinity chromatography using a Capto L resin (GE Healthcare, Little Chalfont, UK) at a 2 ml/min flow rate. The resin was then washed with PBS, and 2E9IgA1 was eluted in 0.1 M glycine pH 2.5, followed by immediate neutralization by addition of TRIS buffer pH 9. Fractions containing protein were then pooled and dialysed using a cassette (Thermo Scientific, UK) against PBS pH 7.4 overnight and concentrated using a 30 kDa cut-off centrifugal concentrator (Amicon-Merck, Watford, UK). Purified 2E9IgA1 was characterized by SDS-PAGE using 4–12% BIS-TRIS gels under non-reducing and reducing (β-mercaptoethanol) conditions. The gels were stained using Coomassie blue (Expedeon, Cambridge, UK) or blotted onto nitrocellulose membranes and subsequently stained using horseradish peroxidase (HRP) conjugated anti-human α or κ chain antibodies (The Binding Site, Birmingham, UK) and developed using ECL Prime (Amersham, UK). Acr binding ELISA was carried out by coating microplates (NUNC, Thermo Fisher Scientific, UK) with 5 µg/ml of antigen and detecting antigen specific IgA using HRP conjugated anti-human α-chain (The Binding Site) secondary antibody. ELISA plates were developed using SigmaFast OPD (Sigma) and absorbance was measured at 450 nm.

### Nebulization of 2E9IgA1

2E9IgA1 was aerosolized by using the commercially available Omron Micro Air U22 electronic mesh nebulizer (Omron, Milton Keynes, UK). 1 ml of 100 µg/ml of 2E9IgA1 in either PBS pH 7.4 alone or PBS containing 0.01% Tween-20 (Sigma) were loaded into the fluid chamber and the exhaust sealed onto a pre-weighed collection vessel at a 45° downward angle. The nebulizer was then turned on and samples left to nebulise until the fluid chamber was emptied. Once completed, the collection vessel was then sealed and placed in ice to condense the nebulized sample. The condensed sample was aspirated to determine protein concentration by OD_280_. Acr and CD89 binding of pre- and post-nebulized samples were determined by ELISA with samples loaded at equal protein concentration.

### Production of 2E9IgG1

Plasmids were expanded and transfected into 293T cells. Cells were plated at a density of 5.0 x 10^6^ cells in 10 ml in a 10 cm dish. 10 ug of total DNA were transfected onto cells using 293 TransIT (Mirus Bio, Madison, US) following manufacturer’s protocols. Cells were washed 16 h post transfection and cultured with FreeStyle™ 293 Expression media (Gibco, Thermo Fisher Scientific). Cell culture supernatants containing protein were harvested at 7 days post transfection. Antibodies within the supernatants were bound to magnetic protein G beads (Merck, St. Louis, MO), washed three times with PBS, eluted from the beads, and neutralized using 1 M Tris, pH 8.0.

### BCG Binding Assay

BCG Pasteur was grown in 7H9 media (Difco, Becton Dickinson) supplemented with glycerol and stocks prepared by sonication (Hielsher UP200St Vialtweeter, Teltow, Germany) at 70% amplitude and 10 W in three 10 s bursts and passing through a 70 µm cell strainer to remove clumps. *Escherichia coli* was grown in standard LB broth. BCG and *E. coli* samples were then re-suspended in FACS buffer consisting of 0.5% w/v BSA (Sigma) in PBS to an OD_600_ of 0.35. IgA was added at concentrations of 100, 20, 4, 0.8, and 0.2 µg/ml and incubated at room temperature for 2 h. For Acr competition assays, 2E9IgA1 concentration was 0.2 µg/ml, and Acr was also added to the samples to final concentrations of 200, 1.6, and 0.064 µg/ml. After washing three times with FACS buffer, samples were incubated with a FITC conjugated anti-human IgA antibody (The Binding Site) for 1 h. Samples were then washed a further three times with FACS buffer and acquired on a BD FACSCalibur cytometer. Data was analyzed using Flowjo V10.

### Persistence of Mucosally Applied 2E9IgA1 in Mice and Legendplex Assay

Human IgA receptor CD89 transgenic BALB/c mice were housed at the St George’s University of London Biological Research Facility and given food and water *ad libitum*. Some animals were inoculated with 0.5 million colony forming units (CFU) of BCG intranasally. Animals were then divided into 2 groups of 9, with the control group receiving 32.5 µl PBS and the 2E9IgA1 group receiving 50 µg of antibody and 1 μg of IFN-γ; per animal in 32.5 µl total volume. All treatments were given by the intranasal route. Three animals were then culled by cervical dislocation at 24, 48, and 72 h post treatment, and bronchoalveolar lavage (BAL) was taken by injecting 1 ml of PBS through the trachea and withdrawing three times. BAL samples were then centrifuged at 14,000 RCF to remove debris. Cytokine levels in the BAL fluid 24 h post-treatment were determined using a Legendplex assay (Biolegend, San Diego, US), acquired on a BD FACSCanto, and subsequently analyzed using the Legendplex software (Biolegend). 2E9IgA1 in the BAL fluid was determined by Acr ELISA, as described previously.

### *In Vitro* Experiments

For studies of mechanisms of 2E9IgA1 modulation of infection, beads or *M. tuberculosis* bacilli were used with human macrophage/monocyte cell lines THP-1 and U937 (ATCC, LGC Standards, Teddington, UK), or neutrophils from blood of healthy volunteers, following ethical approvals. Neutrophils were purified from blood using HetaSep (Stemcell, Cambridge, UK) in a 1:5 ratio and incubated for 30 min to separate leukocytes from erythrocytes. Subsequently, the white-coloured layer of leukocytes was transferred to a fresh tube and neutrophils were isolated using the direct human neutrophil isolation kit (Stemcell) according to manufacturer’s instructions.

#### Neutrophils Phagocytosis

Biotinylated antigen was coupled to yellow-green Neutravidin beads (Life Technologies, Thermo Fisher Scientific). 2E9IgA1 was diluted in culture medium and incubated with antigen-coated beads for 2 h at 37°C. Freshly isolated neutrophils (5x10^4^ cells/well) were incubated for 1 h at 37°C. Cells were stained for CD66b (Clone G10F5; Biolegend), CD3 (Clone UCHT1; BD Biosciences), and CD14 (Clone MP9; BD Biosciences), fixed with 4% paraformaldehyde, and analyzed by flow cytometry. Neutrophils were defined as SSC-A high CD66b+, CD3-, and CD14-. A phagocytic score was determined using the following formula: (percentage of FITC+ cells)*(geometric mean fluorescent intensity (gMFI) of the FITC+ cells)/10,000.

#### Monocyte Phagocytosis

Antigen-coated beads were generated as described for neutrophils. 2E9IgA1 was diluted in culture medium and incubated with antigen-coated beads for 2 h at 37°C. Unbound antibodies were removed by centrifugation prior to the addition of THP-1 cells at 2.5 x 10^4^ cells/well. Cells were fixed with 4% paraformaldehyde and analyzed by flow cytometry. A phagocytic score was determined as described above.

#### Mycobacterial Inhibition Assay

U937 cells (100,000/well) in 96-well plates were infected with *M. tuberculosis* at the multiplicity of infection (MOI) of 2:1. Cultures were supplemented with either PBS, IFN-γ; (20 ng/ml) or 2E9IgA1 (100 μg/ml) and IFN-γ;. Five days later, the cultures were treated for 2 h with 50 μg/ml of amikacin to kill extracellular bacteria, after which the cells were lysed and the intracellular infection quantified by plating on 7H11 Middlebrook agar plates supplemented with OADC (Becton Dickinson). To test the effect of neutrophils on infected macrophages, murine J774 cells in 6-well plates (5 million cells/well) were infected with *M. tuberculosis* at the MOI 2:1, for 24 h. The cultures were then supplemented with human neutrophils isolated from blood of healthy volunteers at the ratio of neutrophils-to-macrophages 5:1. Either PBS or 2E9IgA1 (100 μg/ml) was also added to the cultures and after 24 h, amikacin treatment performed. The cells were then lysed and plated for CFU enumeration, as above.

### Combined Immunotherapy of MDR-TB in Transgenic Mice

All animals were used with approval from St George’s University of London Ethics Committee under an approved UK Home Office animal project licence and used in accordance with the Animals (Scientific Procedures) Act 1986. 3–9-month-old CD89 and CD64 transgenic mice were used, both males and females. Groups consisted of nine animals, following power calculations based on the minimal anticipated magnitude of the effect of 0.5 log difference, 95% statistical power, and an average intra-group variability from previous experiments of 1 log. Mice were housed in containment level 3 facilities at the St George’s University of London Biological Research Facility and challenged with MDR-TB (clinical isolate from Peru, strain number 10095) at 100–200 CFU per animal by the nose-only aerosol system (Biaera Technologies, Hagerstown, MD, US). Infectious dose per animal was determined by sampling airflow during MDR-TB challenge by the use of an impinger and enumeration of sample by CFU. For animals receiving 2E9IgA1 treatment, CIT was given either on the same day as MDR-TB challenge, 24 h or 7 days post-challenge, consisting of 50 µg/animal of 2E9IgA1 and 1 µg/animal of murine IFN-γ per treatment. The treatment regimens for all 2E9IgA1 experiments consisted of three intranasal doses given at three-day intervals after the first treatment. Animals receiving 2E9IgG1 started their treatment regimen 24 h post-challenge. The first dose consisted of 20 µg/animal of 2E9IgG1 + 1 µg/animal of IFN-γ given intranasally. The second dose consisting of 40 µg/animal of 2E9IgG1 + 2 µg/animal of IFN-γ was given intra-peritoneally, 3 days later. After another 3 days, the final dose was given again intranasally, as the first treatment. All experimental animals were culled at 21 days post-challenge, lungs were excised and homogenized in PBS 0.1% w/v Triton X-100 in a Precellys homogeniser (Bertin Instruments, Montigny-le-Bretonneux, France), and serial dilutions were plated in duplicates on 7H11 agar plates to enumerate CFU.

### IgA Detection in TB Patient Sera

Studies on sera samples from the TB patient and their contacts cohort in Mozambique were approved by the Ministry of Health Committee of Bioethics and Health 9ref 298/CNBS/15. Serum samples were collected and sterile filtered using 0.22 µm PES filters (Millex). Antigen binding ELISA was carried out by coating ELISA plates (NUNC) with Acr or MPT64 at 5 µg/ml overnight and detecting antigen specific serum IgA with an HRP conjugated anti-human α-chain antibody (The Binding Site). ELISA plates were developed with SigmaFast OPD (Sigma) and absorbance was read at 495nm.

### Statistical Analysis

All statistical analyses were carried out using Graphpad Prism 8. Multivariance one-way ANOVA was used for multiple comparisons followed by post-hoc group-to-group comparisons as indicated in figure legends. Differences were considered significant when the p value was equal or less than 0.05.

## Results

### 2E9IgA1 Binding Studies

**Figure 1:**
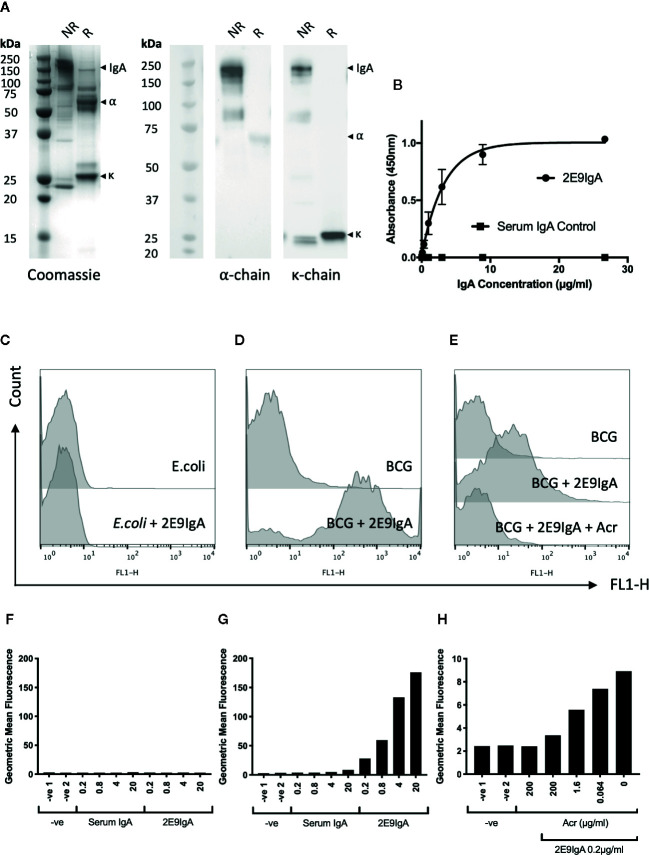
Biophysical characterisation of 2E9IgA1. SDS-PAGE Coomassie and Western blot **(A)** were performed under non-reducing (NR) and reducing (R) conditions. α and κ chains were detected using HRP conjugated anti-human α and κ chain secondary antibodies and developed with ECL prime substrate. 2E9IgA1 binding to purified recombinant Acr antigen was investigated by ELISA **(B)** with error bars indicating SEM. Binding of 2E9IgA1 to **(E)** *coli* **(C**, **F)** and BCG **(D**, **G)** was investigated by flow cytometry using an anti-human IgA (α-chain) FITC conjugated antibody and acquired on a BD FACSCalibur. Antigen specificity of 2E9IgA1 binding to BCG was performed by an Acr competition assay prior to data acquisition by flow cytometry **(E**, **H)**. Experiment performed two times, with similar outcomes.

The 2E9IgA1 mAb was expressed in CHO cells as described previously (10). The purified antibody migrated as a 160 kDa protein band under non-reducing conditions and as 55 kDa (alpha chain) and 25 kDa (kappa chain) protein bands under reducing conditions, as shown by Coomassie staining or Western blot (**Figure 1A**). 2E9IgA1 was fully functional as determined in direct antigen binding assay (**Figure 1B**) and bound specifically to the surface of BCG as confirmed by flow cytometry (**Figures 1D, G**), while no binding could be observed with *E. coli* (**Figures 1C, F**). This binding to mycobacteria was Acr specific since increasing concentrations of soluble Acr antigen progressively inhibited it to near completion (**Figures 1E, H**). Thus, using BCG as a surrogate pathogen, we demonstrate dose dependent surface binding of 2E9IgA1 which could not be observed with *E. coli*.

### Intranasal Inoculation of 2E9IgA1 in Mice

**Figure 2:**
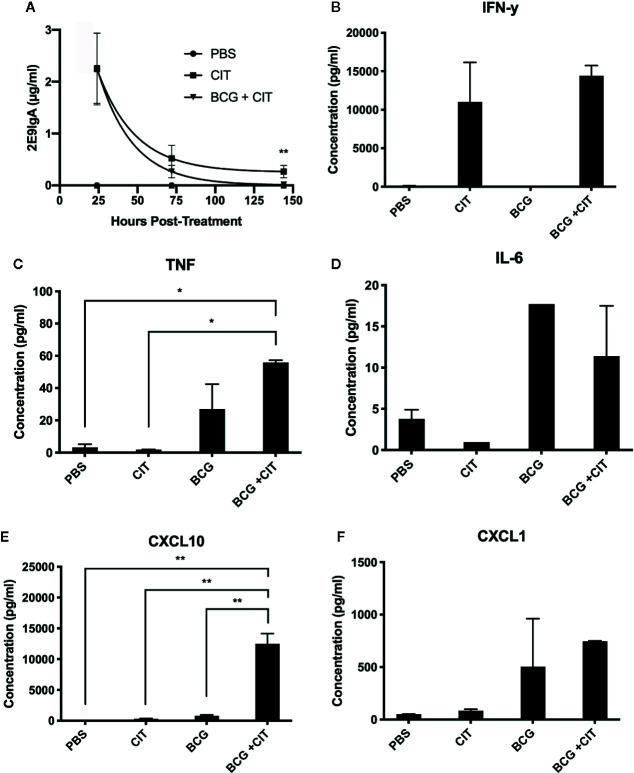
Persistence of nasally applied IgA in mice and cytokine response to CIT in BAL. CD89 transgenic mice were treated with intranasal PBS or CIT either with or without prior intranasal BCG challenge. 2E9IgA1 titres in BAL fluids were determined by ELISA to assess persistence of exogenous 2E9IgA1 in the lungs of mice **(A)**, ** indicates p ≤ 0.01 using students t test. Cytokine levels in BAL fluids were quantified by a multiplex flow-cytometric assay and acquired on a BD FACSCanto to determine IFN-γ **(B)**, TNF **(C)**, IL-6 **(D)**, CXCL10 **(E)**, and CXCL1 **(F)** responses, *p ≤ 0.05, **p ≤ 0.01 using one-way ANOVA with Tukey’s correction. Error bars indicate SEM of three biological samples.

Considering that CIT was to be applied mucosally, we wanted to determine persistence of the 2E9IgA1 mAb following intranasal application to CD89 Tg mice. Mice were inoculated 50 μg of 2E9 mAb and 1 μg of murine IFN-γ; and broncho-alveolar lavage (BAL) recovered at different time points. To determine if CIT alters the lung cytokine milieu following mycobacterial challenge, CIT was also co-applied with BCG (0.5 million CFU). As can be seen in **Figure 2A**, 2E9IgA1 mAb can be recovered from BAL up to 150 h later with the half-life being 75 h. Presence of BCG only marginally decreased the half-life of 2E9IgA1 mAb in the BAL (60 h). We then analyzed selected cytokine and chemokine concentrations in BAL at 75 h post treatment. As expected, we could detect a significant amount of IFN-γ; (**Figure 2B**), which is likely to be largely the exogenously applied IFN-γ;. We also observed an increase in TNF when BCG was co-applied with CIT compared to BCG alone (**Figure 2C**) although this difference was not statistically significant. No statistically significant difference was seen in IL-6 response between BCG and BCG + CIT groups (**Figure 2D**). Interestingly, despite exogenous IFN-γ administration in CIT-treated animals, levels of CXCL10 did not increase significantly compared to PBS-treated animals as expected (**Figure 2E**). The most striking difference, however, was a dramatic increase in the levels of CXCL10 in BCG + CIT BAL compared to BCG alone (**Figure 2E**). We also observed a moderate though not statistically significant increase in CXCL1 levels in the CIT+BCG group compared to those receiving BCG alone (**Figure 2F**).

### Suppression of MDR-TB Infection With 2E9IgA1

**Figure 3:**
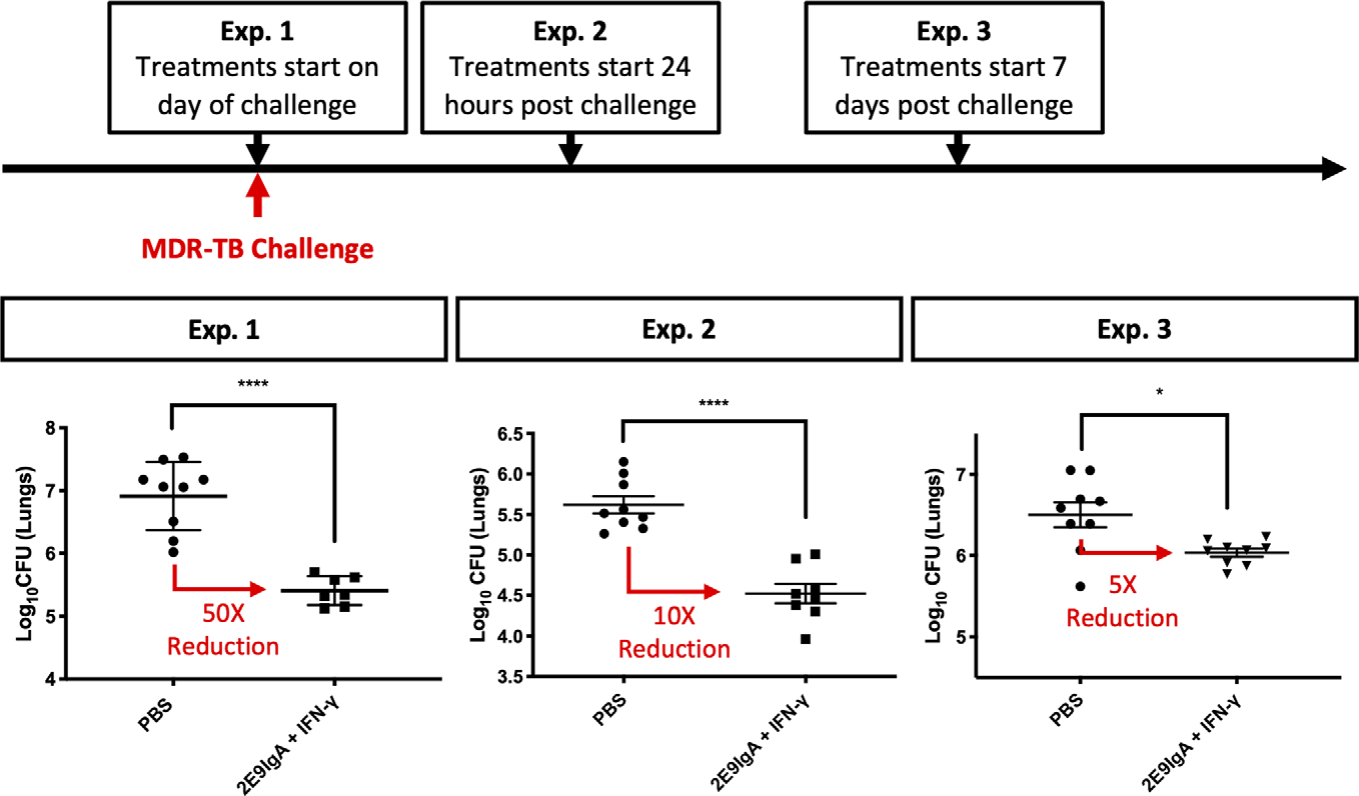
CIT treatment of aerosol MDR-TB challenge in CD89 transgenic mice. CD89 transgenic BALB/c mice were challenged with 100–200 CFU MDR-TB by the nose-only aerosol challenge method. Animals were given treatment of 50 µg/animal 2E9IgA1 + 1 µg/animal IFN-γ (CIT) either on the day of challenge (Exp.1), 24 h post challenge (Exp. 2) or 7 days post challenge (Exp. 3), with the two subsequent intranasal doses at three-day intervals. Animals were culled at 3 weeks post-challenge and lung CFU were enumerated. Error bars indicate SEM. *p ≤ 0.05, ****p0.0001 as determined by one-way ANOVA with Tukey’s correction; n = 8. Three independent experiments.

To test the therapeutic potential of combined 2E9IgA1 and IFN-γ; treatment (CIT), we infected mice with a clinical isolate of MDR-TB by aerosol administration of 100 (exp. 1 and 3) or 200 (Exp.2) CFU. We then tested CIT in separate experiments using three different regimens. The difference was in the start of the treatment in relation to infection: at the time of pathogenic challenge (Exp.1), 1 day after challenge (Exp.2) or 7 days after challenge (Exp.3). In each experiment the treatment was given two more times, 3 and 6 days later and mice culled at 3 weeks. As can be seen in **Figure 3**, all three regimens resulted in a statistically significant reduction of the infection, albeit to a different degree. Thus, initiating CIT at the time of infection gave the greatest (50-fold) reduction in lung CFU, while delaying the start of the treatment to 1 and 7 days resulted in 10- and 5-fold reduction. While we did not perform individual treatment controls in these experiments due to restricted numbers of available CD89 Tg animals, our previous work (10) and results from **Figure 5** (below) showed that while IFN-γ; showed a trend toward protection, it alone was not sufficient to induce a statistically significant lung CFU reduction in any of our experiments. On the other hand, while 2E9IgA1 alone is somewhat protective (10), it combines best with IFN-γ; to induce the observed CIT-mediated suppression of infection in additive or possibly synergistic fashion.

### Mechanisms That May Be Involved in 2E9IgA1 Mediated Protection

**Figure 4:**
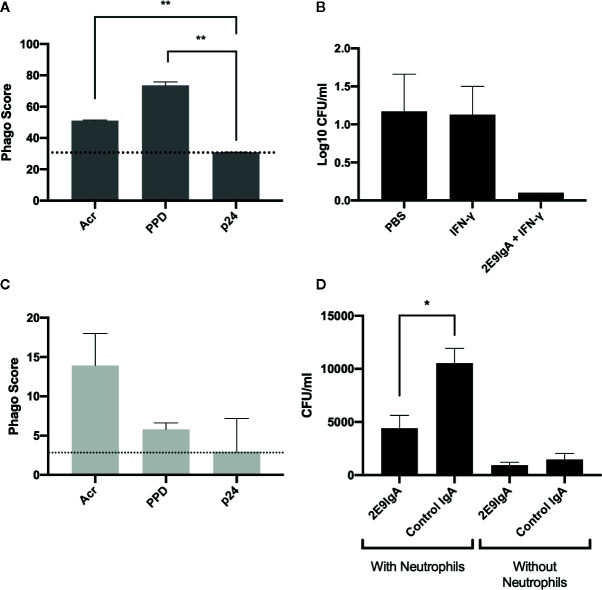
*In vitro* 2E9IgA1 modulation of phagocytic uptake and mycobacterial infection. Macrophage THP-1 cell phagocytic uptake of beads opsonised with Acr or PPD mediated by 2E9IgA1 at 0.5 µg/ml **(A)**. Macrophage U937 monocyte infection with *M. tuberculosis* in the presence or absence of CIT **(B)**; cells were infected for five days and shown are the means + SEM of triplicate cultures. Human neutrophil uptake of beads opsonized with Acr **(C)**. Infection of mouse J774 cells in the presence of 0.1 µg/ml of 2E9IgA1 and human neutrophils **(D)**; cells were infected with *M. tuberculosis* for 24 h and supplemented with neutrophils at a MOI of 5:1 for another 24 h; shown are the means of triplicate cultures + SEM. Neutrophil experiment performed once and macrophage/monocyte twice, with similar outcomes. *p ≤ 0.05, **p ≤ 0.01 as determined by one-way ANOVA with Tukey’s correction.

To elucidate the potential mechanisms that may contribute to 2E9IgA1 mediated protection against MDR-TB infection, we focused on phagocytic uptake and the potential role of neutrophils. First, we demonstrated that 2E9IgA1 promotes phagocytic uptake by human THP-1 monocyte/macrophage cell line (**Figure 4A**) so that Acr and PPD (mycobacterial purified protein derivative) coated beads were taken up more efficiently than HIV-derived p24 coated beads. This increased phagocytic uptake appears to result in a better long-term control (5 days) of intracellular mycobacterial infection, as shown in **Figure 4B**. Similarly, though less efficient than macrophages, human neutrophils also showed enhanced phagocytic uptake of Acr and PPD-coated beads (**Figure 4C**). To elucidate the potential role for neutrophils in IgA-mediated inhibition of infection, we infected mouse macrophage J774 cells with *M. tuberculosis* strain H37Rv in the presence of 2E9IgA1 or a control IgA, and supplemented the cultures with human neutrophils for 24 h. Like macrophages, human neutrophils strongly express CD89 receptor but their role in TB immunity is insufficiently described. As can be seen in **Figure 4D**, the presence of neutrophils (5:1 ratio to macrophages) appeared to paradoxically increase the level of cell infection in this culture system and this is likely due to neutrophils themselves becoming infected or at least harbouring partially digested but still live organisms. Nevertheless, while 2E9IgA1 did not show statistically significant reduction of J774 cell infection, there was a difference over control IgA in the presence of neutrophils. The limitation in this experiment is the short half-life of neutrophils and inability to distinguish bacterial uptake between macrophages and neutrophils; nevertheless, these findings seem to indicate that neutrophils may indeed play a role in a more complex *in vivo* environment and that they may contribute to the therapeutic effect of CIT in CD89 transgenic mice.

### Lack of Protection by the 2E9IgG1 Anti-Acr mAb

**Figure 5:**
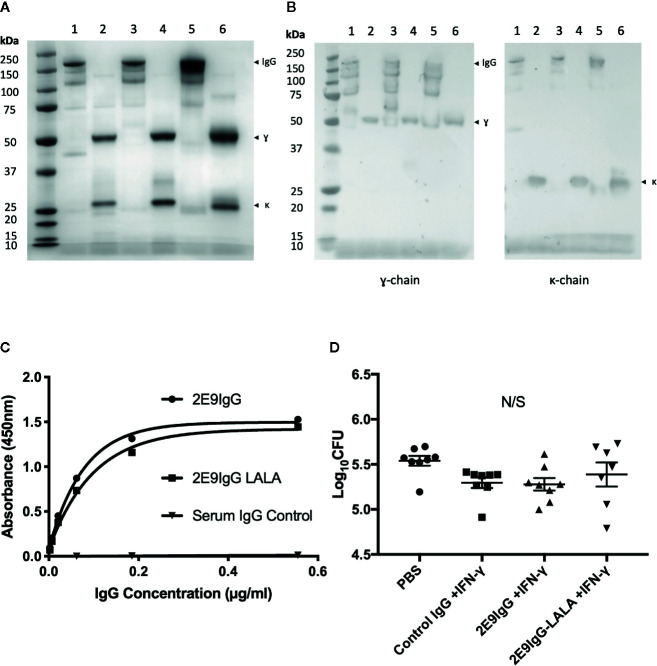
Effect of 2E9IgG1 on aerosol MDR-TB challenge in CD64 transgenic mice. Recombinant 2E9IgG1 was characterized by SDS-PAGE Coomassie **(A)** and Western blot **(B)** using HRP conjugated anti-human IgG (γ-chain) secondary antibody. SDS-PAGE loading order was as follows: 1) 2E9IgG1 (NR), 2) 2E9IgG1 (R), 3) 2E9IgG1-LALA (NR), 4) 2E9IgG1-LALA (R), 5) human serum IgG (NR), and 6) Human Serum IgG (R). 2E9IgG1 and 2E9IgG1-LALA binding to Acr was investigated by ELISA **(C)**. CD64 transgenic BALB/c mice were challenged with 200 CFU MDR-TB by the nose only aerosol method, and treated with three doses of 2E9IgG1 + IFN-γ. The first dose consisted of 20 µg/animal of 2E9IgG + 1 µg/animal of IFN-γ given intranasally. The second dose was given by the intraperitoneal route and consisted of 40 µg/animal of 2E9IgG1 + 2 µg/animal of IFN-γ. The third dose was given intranasally, as the first. Animals were culled 21 days after the MDR-TB challenge and lung CFUs were enumerated **(D)**. Error bars indicate SEM. N/S indicates no significance by one-way ANOVA with Tukey’s correction; n = 8. Mouse experiment performed once.

Since effector functions of FcR gamma and alpha antibody chains differ significantly, we next wanted to test if the IgG version of the same antibody would also be protective. If so, this could be a potential advantage in translation of this approach to clinical treatment of MDR-TB, since unlike IgA, there are numerous licensed IgG-based treatments against various conditions (21, 22). In addition to a fully functional 2E9IgG1, we also generated a mutant of the mAb (LALA) which cannot bind to human Fc-gamma receptor. We tested these two versions of 2E9IgG1 mAb in transgenic mice expressing the human Fcγ;R1 receptor (CD64). This being an IgG, we modified the treatment regimen to include one bolus peritoneal injection, preceded and followed by an intranasal administration, again in combination with IFN-γ;. We also used a control IgG1 (Sigma) as an additional negative control. As can be seen in **Figure 5**, none of the treatments induced statistically significant reduction of lung CFU, although a trend toward reduction compared to no treatment control (PBS) was observed. While the treatment regimen was modified in this experiment and a different strain of transgenic mice was used, we took these outcomes to mean that IgA exerts superior therapeutic potential over IgG under the specific experimental conditions in our studies.

### Detection of Anti-Acr IgA Antibodies in TB Patients and Healthy Controls

We expanded our analysis to humans, by testing the levels of IgA antibodies in sera of TB patients. Anti-Acr IgA ELISA was performed on serum samples from 21 TB patients, 17 exposed contacts, and 19 uninfected healthy controls from a study cohort in Maputo, Mozambique. All participants were vaccinated with BCG at birth as per national guidelines, and this could be confirmed by a visible scar in the majority. We also tested these sera for antibodies against the virulence associated factor MPT-64 antigen.

**Figure 6:**
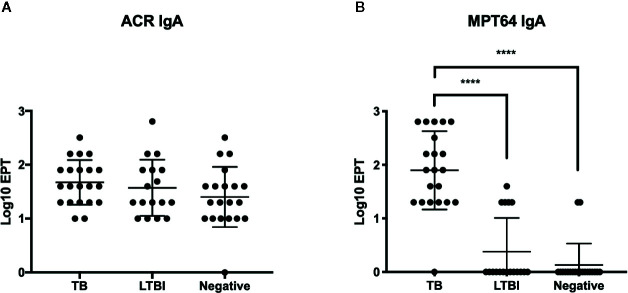
IgA responses in patient serum samples from Instituto Nacional de Saúde, Mozambique. End point titres (EPT) (defined as 2-fold absorbance value of background samples) of IgA in serum against Acr **(A)** and MPT64 **(B)** were determined by ELISA with error bars indicating SEM. ****p ≤ 0.0001, one-way ANOVA with Tukey’s correction; n = 21 (TB patients), 17 (LTBI), and 19 (BCG vaccinated). Experiment performed once.

Serum IgA antibody levels (**Figure 6**) to Acr were well demonstrable and notably, and they were found to be of similar magnitude in all three tested groups. In sharp contrast, IgA response to MPT-64 strongly elevated in active TB patients, while the LTBI individuals and uninfected controls showed no responses apart from a small proportion of participants who displayed a weak response.

### Assessing the Feasibility of Aerosol Delivery for 2E9IgA

**Figure 7:**
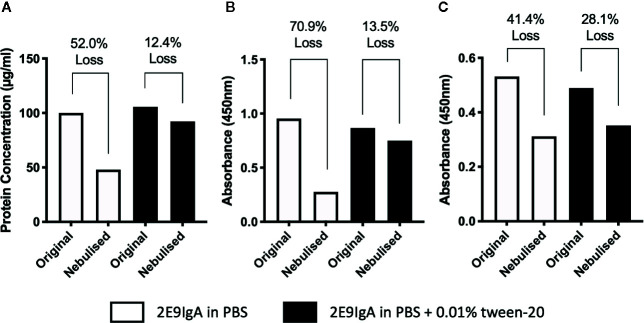
Assessing the feasibility of aerosolized delivery of 2E9IgA1. 1 ml of 100 µg/ml of 2E9IgA1 was nebulized using an Omron MicroAir U22 portable mesh nebulizer, either in PBS alone or with 0.01% Tween-20. Condensate was collected, protein was recovered **(A)**, and Acr binding by ELISA **(B)** and recombinant CD89 binding activity by ELISA **(C)** were investigated to compare original and nebulized samples with equal protein loading. Experiment performed twice, with similar results.

With a view to move toward initiating human clinical trials for CIT, a delivery system suitable for human application must be considered. To that end, we explored the use of aerosolization to deliver 2E9IgA to mucosal surfaces using the widely available Omron Micro Air nebuliser. Antibodies are bioactive, relatively large molecules, and the process of aerosolization in a nebulizer may lead to loss of protein and/or activity (23). Indeed, when we aerosolized 2E9IgA1, only 48% of the protein was recovered in the condensate (**Figure 7A**) and this associated also with a significant loss of antigen-binding capacity (**Figure 7B**) and receptor coupling of the recovered protein (**Figure 7C**). However, these losses were largely reversed by adding 0.01% Tween-20 (Polysorbate-20) to the antibody preparation so that only 12.4% antibody was lost, coupled with a 13.5% loss of antigen binding and 28.1% loss of receptor binding. These findings suggest that aerosolized delivery of 2E9IgA using the Omron nebulizer is feasible provided that a non-ionic detergent is included in the formulation, although there is further scope for optimization.

## Discussion

The present study demonstrated that CIT could suppress MDR-TB infection in mice bearing the human IgA receptor. As demonstrated in three different experimental scenarios, the most significant therapeutic effect was observed when CIT treatment was initiated at the time of infection. Importantly though, we observed a significant reduction in lung CFU even when the infection was allowed to establish, at 1-week post-exposure. The mandatory role of the IgA isotype was suggested by the lack of protection by the IgG version of 2E9IgA1 in Fc-gamma receptor transgenic mice. Furthermore, our previous work has shown that protection against *Mtb* challenge in 2E9IgA-treated mice was dependent on mice being transgenic for the CD89 receptor (10). CD89 expression in transgenic mice was previously detected on neutrophils, monocytes and weakly positive on macrophages (24) which was similar to the expression profile in found in humans (25). Given our understanding for the different functional roles of IgA and IgG, these data imply that the immune-function of IgA provides superior control over *M. tuberculosis* compared to those exerted by IgG, although differences in glycosylation between the two antibody isotypes tested may also play a role. The superiority of IgA over IgG has also been reported in other studies (5), but the present experiments are the first to compare the two isotypes using antibodies of the same epitope specificity. This IgA phenomenon may be most important because of the mucosal nature of TB, where the innate immune cells in the lung may be heavily involved in the bacterial clearance.

The potential mechanisms involved were ascertained by the demonstration that beads opsonised with 2E9IgA1 underwent enhanced phagocytosis by macrophages and neutrophils. Antibody mediated phagocytosis is known to enhance phago-lysosomal fusion (26), circumventing the pathogen mediated inhibition of this process, a prominent survival mechanism used by *M. tuberculosis* (27). Interactions with antibody FcR and TLRs (28, 29) activate host defence mechanisms while interactions with complement receptors favour bacterial survival (30). FcγR interaction activates signal transduction that increases macrophage Ca^2+^ influx and subsequent intracellular killing (31). Since 2E9IgA1 has been shown to improve bacterial uptake by monocyte/macrophages, it is possible that this may lead to more efficient clearance of organisms by phago-lysosomal killing mechanisms, especially when combined with macrophage activating IFN-γ;. We also found that 2E9IgA1 improves bacterial control in the presence of neutrophils although the overall level of infection in this two-cell type co-culture system is paradoxically increased. This was most probably due to some of the bacteria being ingested by the neutrophils themselves but not yet killed. Further studies are required to elucidate the role of neutrophils in *M. tuberculosis* infection and the potential role of antibodies in their mode of action.

Targeting of 2E9IgA1 to the 16kDa Acr antigen may be an important determinant of its protective potential. Acr is prominently expressed by *M. tuberculosis* inside macrophages, under hypoxia-induced stress conditions (32), and is also associated with dormancy (33). While its expression is mostly confined to cytosol, it can be also found associated with cell wall (33). For the purpose of our study, it was important to demonstrate the availability of Acr antigen on the surface of mycobacteria, as a prerequisite for 2E9IgA1 mAb therapeutic activity. Indeed, we showed that 2E9IgA1 could bind efficiently and in a dose-dependent manner to BCG surface and that this could be inhibited with soluble Acr. Thus, this establishes the Acr antigen as a novel target for immunotherapy and is suggestive of possibly also other non-classical cell wall proteins that could be targeted by antibody treatments.

Evidence gathered from this and other studies points toward beneficial effects from antibody-based combined immunotherapies which could be exploited in conjunction with chemotherapy (9, 34–38). Screening of the current and new antibiotics with monoclonal antibodies may represent an untapped opportunity in the setting of ever-increasing microbial drug resistance.

The field should now move toward the study of recombinant human antibodies, with a view to initiating clinical trials. With that in mind, we performed initial aerosolization studies with 2E9IgA1, to test the feasibility of this concept. Aerosolized, lung-targeted delivery would be the most appropriate mode of delivery of CIT as an adjunctive treatment of MDR-TB. Previous clinical studies have demonstrated feasibility of aerosolized delivery of IFN-γ; to MDR-TB patients (39, 40) but no such evidence exist for 2E9IgA1, or antibodies in general. We demonstrated that with the addition of 0.01% Tween-20 into the antibody sample, protein recovery, antigen binding activity and host Fc receptor binding activity can largely be recovered from the condensate. Tween-20 (and Tween-80, which showed similar results) is permissible for human application in low concentrations and is used in foods and medications as a dispersant or excipient (41, 42). These results demonstrated the feasibility of 2E9IgA1 aerosolized delivery and warrant further optimisation studies to minimise these losses even further.

Testing serum IgA antibodies showed elevated anti-Acr levels in LTBI and BCG vaccinated subjects. This finding is in accord with the previous report that occupational TB exposure associated with elevated antibody levels to Acr, but not to other immunodominant antigens (43). Interestingly, another recent study reported an increase of IgM anti-Acr antibodies in sera of LTBI individuals (44) but that study did not include analysis of IgA. On the other hand, the lack of further increase of anti-Acr IgA levels in sera of active TB patients can be interpreted as IgA-class selective, since the increase of total antibodies in TB patients to this antigen can be attributed to anti-Acr antibodies of the IgG isotype (45). The results could be interpreted also with the assumption, that human IgA antibodies directed against Acr epitopes are shared between *M. tuberculosis*, BCG and non-tuberculous mycobacteria. In contrast, anti-MPT64 antibody levels were selectively elevated in active disease, but mostly below detection in LTBI- and BCG-vaccinated subjects. Therefore, antibody profiling in various TB study groups and populations may yield important new evidence for humoral biomarkers of disease and immunity to infection. Likewise, it would be of interest to assess in future studies if antibiotic treatment may see an elevation in Acr-specific IgA as a biomarker of response to therapy and elimination of infection.

In summary, we show that recombinant human IgA-based aerosolized delivery of CIT is feasible and deserves further translation toward its potential contribution to the therapy of MDR-TB. The protective attributes are further supported by the finding of anti-Acr IgA antibodies in sera of LTBI- and BCG-vaccinated individuals.

## Data Availability Statement

The raw data supporting the conclusions of this article will be made available by the authors, without undue reservation.

## Ethics Statement

The studies involving human participants were reviewed and approved by National Bioethics Review Board of Mozambique. The patients/participants provided their written informed consent to participate in this study. The animal study was reviewed and approved by St. George’s University of London Ethics Committee, London, United Kingdom.

## Author Contributions

AT and GD contributed to the work equally. AT, MP, JI, and RR drafted and edited this manuscript. AT, GD, MP, NM, EI, JI, GA, and RR contributed to *in vitro* experiments. AT, GD, MP, AC, PH, and RR contributed to mouse experiments. TM contributed to human cohort sample collection. AT, GD, MP, PD, and JI contributed to statistical analysis. All authors contributed to the article and approved the submitted version.

## Funding

This study was supported by a European Union H2020 grant no. 643558 as part of the Eliciting Mucosal Immunity in Tuberculosis (EMI-TB) consortium project.

## Conflict of Interest

The authors declare that the research was conducted in the absence of any commercial or financial relationships that could be construed as a potential conflict of interest.
